# Acute Myocardial Infarction and Stress Cardiomyopathy following the Christchurch Earthquakes

**DOI:** 10.1371/journal.pone.0068504

**Published:** 2013-07-02

**Authors:** Christina Chan, John Elliott, Richard Troughton, Christopher Frampton, David Smyth, Ian Crozier, Paul Bridgman

**Affiliations:** 1 Department of Cardiology, Christchurch Hospital, Christchurch, New Zealand; 2 University of Otago, Christchurch Campus, Christchurch, New Zealand; Universityhospital Düsseldorf, Germany

## Abstract

**Background:**

Christchurch, New Zealand, was struck by 2 major earthquakes at 4:36am on 4 September 2010, magnitude 7.1 and at 12:51pm on 22 February 2011, magnitude 6.3. Both events caused widespread destruction. Christchurch Hospital was the region's only acute care hospital. It remained functional following both earthquakes. We were able to examine the effects of the 2 earthquakes on acute cardiac presentations.

**Methods:**

Patients admitted under Cardiology in Christchurch Hospital 3 week prior to and 5 weeks following both earthquakes were analysed, with corresponding control periods in September 2009 and February 2010. Patients were categorised based on diagnosis: ST elevation myocardial infarction, Non ST elevation myocardial infarction, stress cardiomyopathy, unstable angina, stable angina, non cardiac chest pain, arrhythmia and others.

**Results:**

There was a significant increase in overall admissions (p<0.003), ST elevation myocardial infarction (p<0.016), and non cardiac chest pain (p<0.022) in the first 2 weeks following the early morning September earthquake. This pattern was not seen after the early afternoon February earthquake. Instead, there was a very large number of stress cardiomyopathy admissions with 21 cases (95% CI 2.6–6.4) in 4 days. There had been 6 stress cardiomyopathy cases after the first earthquake (95% CI 0.44–2.62). Statistical analysis showed this to be a significant difference between the earthquakes (p<0.05).

**Conclusion:**

The early morning September earthquake triggered a large increase in ST elevation myocardial infarction and a few stress cardiomyopathy cases. The early afternoon February earthquake caused significantly more stress cardiomyopathy. Two major earthquakes occurring at different times of day differed in their effect on acute cardiac events.

## Introduction

On 4 September, 2010, Christchurch, the second largest city in New Zealand with an urban population of 400,000, was struck by a 7.1 magnitude earthquake at 4:46 am. The city suffered significant damage to its infrastructure and buildings. A state of emergency was declared and financial loss was estimated to be as high as $5 billion. Fortunately, there were no direct fatalities. Six months and thousands of mild to moderate aftershocks later, Christchurch was again struck by a powerful earthquake on 22 February 2011 at 12:51 pm. Although smaller on the Richter scale than the first one, the epicentre of this magnitude 6.3 earthquake was 5 kilometres deep and 10 kilometres southeast of the city centre. It generated a vertical 2.2G force, the highest peak ground acceleration ever recorded in the world [1]. As many as 185 people died with many more injured and left homeless. More than one third of the buildings in the central business district were destroyed, including 2 multi-story buildings.

Major stressful events are well documented to increase the incidence of acute cardiac events [2]. Cardiovascular complications more than doubled during the FIFA World Cup games of 2006 [3]. After the September 11 terrorist attacks, significantly more patients presented with acute myocardial infarction to the hospitals in Brooklyn [4] and New Jersey [5]. Earthquakes are amongst the most catastrophic natural disasters, and are known to cause adverse cardiovascular events [6]. To make matter worse, mass destruction of organizations and infrastructures can disrupt the health systems' ability to function. In the past, several studies have been done in different countries to show the effects of earthquakes on cardiovascular events [7]–[13]. The results were variable. Fortunately, the Cardiology Department of Christchurch Hospital, the only acute cardiac service provider in the region remained fully functional after both major earthquakes [14]. The study was commenced after the first earthquake with the goal of providing current first world data with complete single centre capture. After the second earthquake the study was extended with the aim of comparing the impact of 2 separate major earthquakes on acute cardiovascular events.

## Methods

### Ethics Statement

The Southern Health and Disability Ethics Committee was consulted for consideration of expedited ethical approval. They advised ethical approval was not required because this study did not require direct patient contact and would not identify individuals.

Within hours of the September 2010 event, acute Cardiology admitting staff noted an increased number of chest pain presentations precipitated by the earthquake and its immediate aftershocks. Faced with this, they maintained a log of all patients who reported that their chest pain was triggered by the earthquake or one of the immediate aftershocks. An audit was then commenced 4 weeks after the earthquake to fully document the pattern of cardiac admissions. Clinical notes and electronic discharge summaries were systematically reviewed and relevant data collected. Following the second earthquake in February 2011 a second review was performed using the same methodology as the first. This has allowed for stringent comparison of the effects of the 2 events.

The electronic patient management system at Christchurch Hospital was used to identify all acute admissions to the Cardiology Department in the period of 3 weeks prior to and 5 weeks following each earthquake (from 14 August to 8 October, 2010 and from 1 February to the 28 March, 2011). The control groups were all acute Cardiology admissions in the corresponding time period from the previous year (from 15 August to 9 October, 2009 and from 2 February to 29 March, 2010). Patients who were not residents of the earthquake zone (Christchurch city, Waimakariri and Selwyn district) were excluded from the study as were peripheral hospital transfers and elective admissions.

Systematic review of the clinical files, electronic discharge summaries, coronary angiograms and echocardiograms were undertaken for each patient by a senior investigator. Data collected included basic patient characteristics, date of admission and discharge, and investigations performed. Cardiac diagnosis was defined according to set criteria.

Cardiac arrhythmia presentations were classified into heart block (second degree or complete), ventricular arrhythmia, atrial fibrillation, atrial flutter or supraventricular tachycardia. Cardiac arrest was defined as arrhythmia (ventricular or asystole) that required electrical cardioversion or CPR to support circulation. All diagnoses of arrhythmia had to be confirmed by review of the ECGs.

ST elevation myocardial infarction (STEMI) was defined as chest pain associated with at least 2 millimetres of ST segment elevation in 2 precordial leads or 1 millimetre in 2 other contiguous leads on ECG. Non ST elevation myocardial infarction (NSTEMI) was defined as chest pain without associated ST segment elevation. Other focal ECG changes such as ST segment depression may or may not be present. For both STEMI and NSTEMI diagnoses a serial troponin I rise to over 0.03 µg/l and a culprit lesion identifiable on coronary angiography were required.

Unstable angina was defined as ischaemic symptoms which can be frequent and onset with little or no physical exertion, with or without dynamic ECG changes, and serial troponin I of<0.03 µg/l. Stable angina was defined as ischaemic symptoms onset only with physical activities, without dynamic ECG changes and serial troponine I of<0.03 µg/l. For newly presented patients, the diagnosis is made following either positive stress tests or coronary angiography to confirm coronary artery disease. For both conditions, in those who have known coronary artery disease on previous coronary angiography, further stress test or repeat imaging was not necessary to make the diagnosis.

Non-cardiac chest pain (NCCP) was defined as chest pain presentation without associated ECG changes and with serial troponin I<0.03 µg/l at least 12 hours after the onset of symptoms. Other significant causes of chest pain had to be excluded to the satisfaction of the attending Cardiologist. Where exercise ECG, exercise Echo or Dobutamine stress Echocardiograms were undertaken, the results were negative. In cases where coronary angiography was performed, significant coronary artery disease was excluded for the patient to be classified as having NCCP.

Stress cardiomyopathy (SCM) was defined similar to the modified Mayo criteria [7]. All patients were admitted with chest pain with evolving ECG changes, a troponin I rise>0.03 µg/l, a recognised transient echocardiographic regional wall motion abnormality (apical ballooning pattern, mid wall variant or basal segment variant), and no culprit lesion on coronary angiography.

Patients presented with heart failure had symptoms and signs in keeping with the diagnosis, plus one of the following – radiographic changes of fluid overload, elevated BNP, or ventricular dysfunction on Echocardiogram. A diagnosis of pericarditis was made if patient had typical symptoms and saddle shaped ST elevation on ECG, with normal serial troponin I (<0.03 µg/l), with or without pericardial effusion on echocardiogram and prodromal illness. Significant valvular heart disease was diagnosed when patients have severe valvular lesion on echocardiography and associated symptoms. The diagnosis of pulmonary embolus was made radiologically either by CT pulmonary angiography or ventilation perfusion scanning.

For each earthquake statistical analysis was performed for total cardiac admissions and for each specific cardiac diagnosis. The change in admission rate from the 3 weeks prior to each earthquake to the 2 weeks after was compared with the relevant control period. A poisson regression model which included terms for the pre and post-earthquake periods, the earthquake and control periods, and the interaction of these 2 factors was used to compare these changes. The interaction term which directly compares the changes between the control and earthquake periods was the focus of the analyses. A two-tailed p-value<0.05 was taken to indicate statistical significance.

## Results

**Figure 1:**
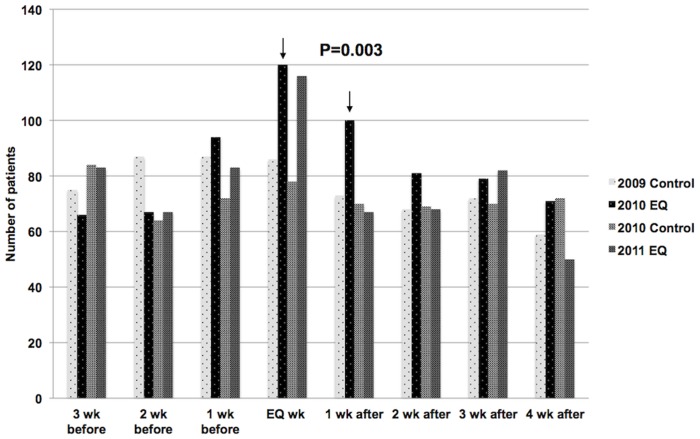
Total acute cardiology admissions.

The pattern of acute cardiology admissions in the weeks before and after the 2 earthquakes is shown in figure 1. On average, 75 patients were acutely admitted each week during the control periods. In the week of the September 2010 event, there were 120 acute admissions. In the following week, there were 100. In the week of the February 2011 event, there was a similar increase to that of the September earthquake, but the admission rate then returned to baseline in the following week. Statistical analysis showed that the increase in total cardiology admissions in the 2 weeks following the first earthquake was significant, p = 0.003 (figure 1).

**Figure 2:**
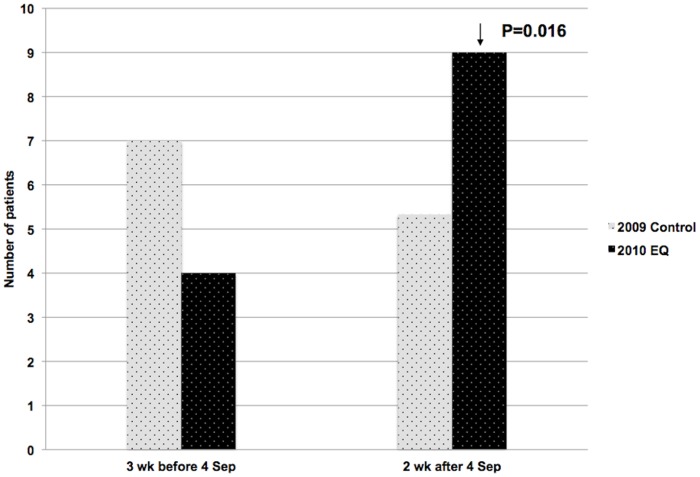
Mean STEMI admissions 3 weeks before and 2 weeks after the September 2010 earthquake.

In the first 2 weeks after the September 2010 earthquake, there was a significant increase in the mean admission rate for STEMI (p = 0.016) compared to that of the control period (3 weeks before and 2 weeks after 4 September 2009), and to 3 weeks prior to the earthquake (figure 2).

**Figure 3:**
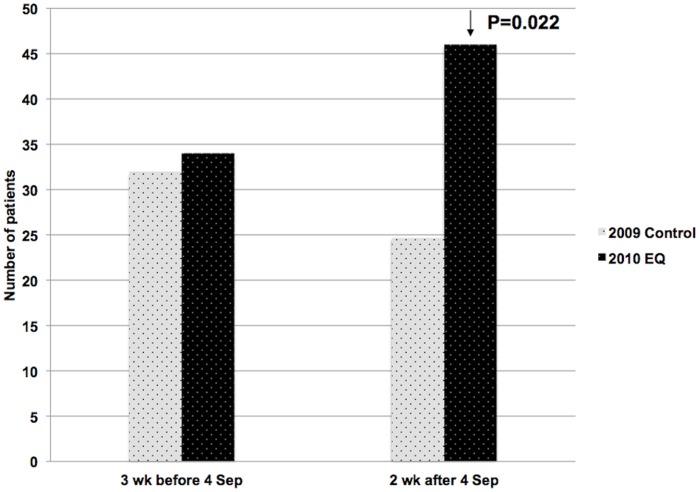
Mean NCCP admissions 3 weeks before and 2 weeks after the September 2010 earthquake.

**Figure 4:**
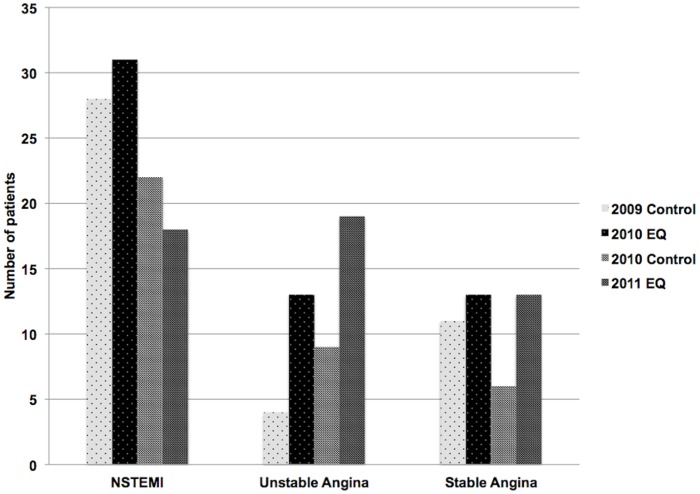
Other chest pain presentations in the first 2 weeks following both earthquakes.

Similarly, there were significantly more NCCP admissions in the first 2 weeks following the September 2010 earthquake, p = 0.022 (figure 3). Amongst patients with a final diagnosis of NCCP 54% underwent noninvasive stress testing and 12% underwent coronary angiography. Rates for other chest pain presentations were not significantly increased (figure 4).

**Figure 5:**
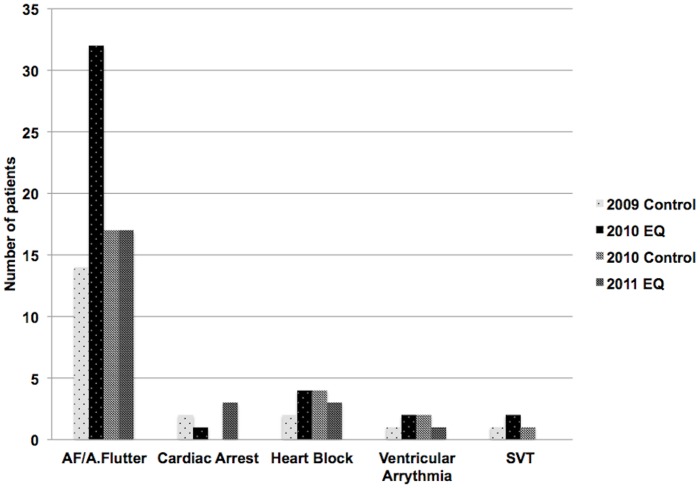
Arrhythmia presentations in the first 2 weeks following both earthquakes. AF  =  atrial fibrillation; A.Flutter  =  atrial flutter; SVT  =  supraventricular tachycardia.

There were no statistically significant changes in acute cardiac arrhythmia presentations in the first 2 weeks following both earthquakes (figure 5), including atrial fibrillation and atrial flutter, p = 0.10.

**Figure 6:**
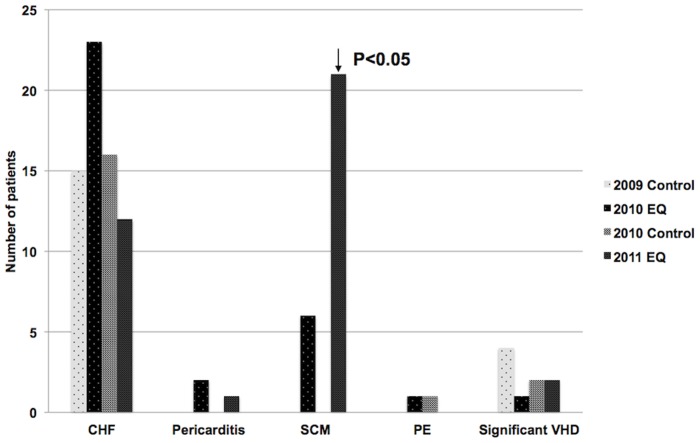
Other cardiac presentations in the first 2 weeks following both earthquakes. CHF  =  congestive heart failure; PE  =  pulmonary embolus; VHD  =  valvular heart disease.

There were no reported cases of SCM in the 2009 and 2010 control periods. After the September earthquake there were 6 cases (6 women, mean age 72 years). There was a dramatic increase with 21 SCM presentations within 4 days of the February 2011 earthquake (21 women, mean age 68 years). The 95% confidence intervals on the rate over 5 weeks are 0 to 1.27 for the control period and 0.44 to 2.62 and 2.6 to 6.4 respectively for each earthquake. Statistical analysis showed this to be a significant difference between the earthquakes (p<0.05). The February 2011 earthquake resulted in significantly more SCM cases than the September 2010 earthquake. Neither earthquake affected presentation rates for congestive heart failure, pericarditis, pulmonary embolus, or significant valvular heart disease (figure 6).

## Discussion

The most striking finding of this study is that the 2 earthquakes differed in their effects on acute cardiac admission pattern. This was most apparent in the first 2 weeks. After that, the changes in acute cardiac admission dissipated. The 2 major earthquakes struck Christchurch within 6 months of each other and each was associated with a different pattern of cardiac admissions. In the week of the September 2010 earthquake and the week following, there was an increase in acute STEMI presentations and a lesser increase of SCM. In the week of the second earthquake, there were 3 times as many SCM cases within 4 days, without a spike in acute myocardial infarction. The pattern following the first earthquake is similar to that reported after the 1994 Northridge earthquake [8]. There was a 35% rise in myocardial infarction presentations in the week following Northridge. A similar sharp increase in the rate of myocardial infarction was reported in the week following the Hanshin-Awaji earthquake of 1995 [9], [10]. In contrast, no increase in myocardial infarction rate was reported following the Loma Prieta earthquake [11]. Loma Prieta occurred at 5:04pm. Christchurch's second earthquake which also did not result in an increase in myocardial infarction occurred at 12:51pm. It is well known that circadian variation exists for myocardial infarction and sudden cardiac death. The risk of an acute cardiac event increases during the morning after waking and arising [15], [16]. Our data and that from these other studies suggest that natural disasters occurring late in the morning and in the afternoon are much less likely to precipitate myocardial infarction than those occurring in the early hours of the morning. The 4 earthquakes with documented increases in acute myocardial infarction rates are the first Christchurch earthquake at 4:36am, Northridge at 4:30am, Hanshin-Awaji at 5:46am and Newcastle at 10:27am [12].

Our data also raises the possibility of circadian variation in propensity to SCM. The earthquake at 12:51pm in February 2011 was smaller than that at 4:36am in September 2010 but resulted in 3 times more cases. The only study specifically examining such condition after an earthquake was from Watanabe et al after the Niigata earthquake in 2004 [13], [17]. That earthquake occurred at 13:01pm. They found 25 cases of SCM in the 4 weeks after the event compared with only 1 case reported in the 4 weeks previously and none in the 2 years before.

Earthquakes are unheralded events. As with previous studies our study has the weakness that by necessity it is retrospective. It's strength however is that it is a single centre experience. Christchurch Hospital is the only acute hospital for the region, and that we have the same population exposed to 2 different earthquakes in a 6 month time period. There was no significant migration in or out of Christchurch between the earthquakes. Furthermore, Christchurch Hospital and our patient management systems remained fully functional in spite of the disruption to the rest of the city, ensuring good data capture.

The major implication of this study for clinicians and policymakers is that each earthquake, and by extrapolation each natural disaster, is different. Christchurch Hospital was fortunate to have effective natural disaster procedures in place prior to these earthquakes. These permitted the Cardiology service to remain functioning throughout and allowed for provision of continued normal care. In our study, the majority of earthquake induced acute cardiac presentations occurred within the first 2 weeks following the events. Such knowledge allows better future disaster planning, focusing on efficient resource and personnel allocation in the initial period following a catastrophe.

It is unclear from this study why SCM might have a diurnal variation. Variations in autonomic tone and haemostatic factors have been proposed as possibly important in the mechanism in myocardial infarction. Following the Hanshin-Awaji earthquake increases in D-dimer, von Willebrand factor, tissue-type plasminogen activator and blood pressure were reported [18]. It is possible that neurologic or autonomic factors may be important in SCM. Prospective studies of earthquakes and cardiac events are of course impossible. Only the largest of earthquakes precipitate myocardial infarction and SCM. None of the 20 or so magnitude 5 to 6 aftershocks to have struck Christchurch have been large enough to cause a single case of SCM or a rise in chest pain presentation rate. The 2 earthquakes we report here are unusual in that they were large enough to precipitate cardiac events but not so large as to cripple the operation of the region's single acute hospital. We were very fortunate indeed.
